# Redetermination of (d-penicillaminato)lead(II)

**DOI:** 10.1107/S1600536812011877

**Published:** 2012-03-28

**Authors:** Adam C. Schell, Masood Parvez, Farideh Jalilehvand

**Affiliations:** a Department of Chemistry, The University of Calgary, 2500 University Drive NW, Calgary, Alberta, Canada T2N 1N4

## Abstract

In the title coordination polymer, [Pb(C_5_H_9_NO_2_S)]_*n*_ {systematic name: *catena*-poly[(μ-2-amino-3-methyl-3-sulfido­butano­ato)lead(II)]}, the d-penicillaminate ligand coordin­ates to the metal ion in an *N*,*S*,*O*-tridentate mode. The S atom acts as a bridge to two neighbouring Pb^II^ ions, thereby forming a double thiol­ate chain. Moreover, the coordinating carboxyl­ate O atom forms bridges to the Pb^II^ ions in the adjacent chain. The overall coordination sphere of the Pb^II^ ion can be described as a highly distorted penta­gonal bipyramid with a void in the equatorial plane between the long Pb—S bonds probably occupied by the stereochemically active inert electron pair. The amino H atoms form N—H⋯S and N—H⋯O hydrogen bonds, resulting in a cluster of four complex units, giving rise to an *R*_4_^4^(16) ring lying in the *ab* plane. The crystal structure of the title compound has been reported previously [Freeman *et al.* (1974 ▶). *Chem. Soc. Chem. Commun.* pp. 366–367] but the atomic coordinates have not been deposited in the Cambridge Structural Database (refcode DPENPB). Additional details of the hydrogen bonding are presented here.

## Related literature

For an earlier characterization of the title compound, see: Freeman *et al.* (1974 ▶). For neurotoxic effects of Pb, see: Needleman (2004 ▶); Bressler *et al.* (1999 ▶); Godwin (2001 ▶). For treatments of lead(II) poisoning, see: Sinicropi *et al.* (2010) ▶; Casas & Sordo (2006 ▶). For graph-set notation, see: Bernstein *et al.* (1994 ▶). 
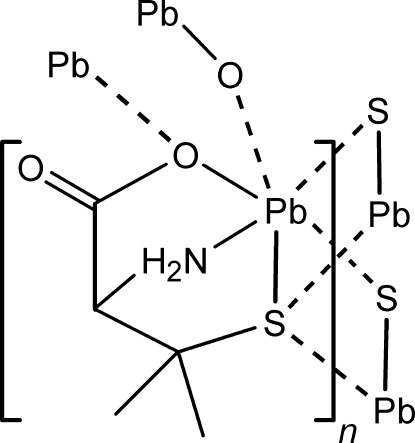


## Experimental

### 

#### Crystal data

[Pb(C_5_H_9_NO_2_S)]
*M* *_r_* = 354.38
Monoclinic, 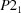

*a* = 6.251 (4) Å
*b* = 6.179 (3) Å
*c* = 10.259 (6) Å
β = 107.72 (2)°
*V* = 377.5 (4) Å^3^
*Z* = 2
Mo *K*α radiation
μ = 22.56 mm^−1^
*T* = 123 K
0.06 × 0.05 × 0.02 mm

#### Data collection

Nonius KappaCCD diffractometer
Absorption correction: multi-scan (*SORTAV*; Blessing, 1997 ▶) *T*_min_ = 0.345, *T*_max_ = 0.661
6589 measured reflections
2157 independent reflections
2027 reflections with *I* > 2σ(*I*)
*R*_int_ = 0.059

#### Refinement

*R*[*F* ^2^ > 2σ(*F* ^2^)] = 0.032
*wR*(*F* ^2^) = 0.085
*S* = 1.09
2157 reflections
93 parameters
1 restraint
H-atom parameters constrained
Δρ_max_ = 2.76 e Å^−3^
Δρ_min_ = −3.17 e Å^−3^
Absolute structure: Flack (1983 ▶), 966 Friedel pairs
Flack parameter: 0.03 (2)

### 

Data collection: *COLLECT* (Hooft, 1998 ▶); cell refinement: *DENZO* (Otwinowski & Minor, 1997 ▶); data reduction: *SCALEPACK* (Otwinowski & Minor, 1997 ▶); program(s) used to solve structure: *SHELXS97* (Sheldrick, 2008 ▶); program(s) used to refine structure: *SHELXL97* (Sheldrick, 2008 ▶); molecular graphics: *ORTEP-3 for Windows* (Farrugia, 1997 ▶); software used to prepare material for publication: *SHELXL97*.

## Supplementary Material

Crystal structure: contains datablock(s) global, I. DOI: 10.1107/S1600536812011877/hb6608sup1.cif

Structure factors: contains datablock(s) I. DOI: 10.1107/S1600536812011877/hb6608Isup2.hkl

Additional supplementary materials:  crystallographic information; 3D view; checkCIF report

## Figures and Tables

**Table 1:** Selected bond lengths (Å)

Pb1—N1	2.444 (9)
Pb1—O1	2.451 (7)
Pb1—S1	2.714 (2)
Pb1—O1^i^	2.719 (7)
Pb1—S1^ii^	3.091 (3)
Pb1—S1^iii^	3.465 (3)

Symmetry codes: (i) 
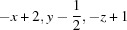
; (ii) 
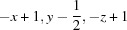
; (iii) 
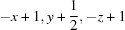
.

**Table 2:** Hydrogen-bond geometry (Å, °)

*D*—H⋯*A*	*D*—H	H⋯*A*	*D*⋯*A*	*D*—H⋯*A*
N1—H1*B*⋯S1^iii^	0.92	2.59	3.453 (8)	156
N1—H1*A*⋯O2^iv^	0.92	2.24	3.070 (10)	150

Symmetry codes: (iii) 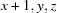; (iv) 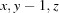.

**Fig. 1.**
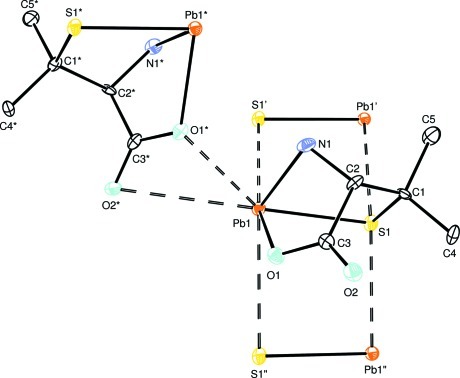
The molecular structure of the title compound. Displacement ellipsoids are drawn at the 50% probability level. H atoms were omitted for clarity. [Symmetry codes: * -x + 2, -1/2 + y, -z + 1; ' -x + 1, -1/2 + y, -z + 1; " -x + 1, 1/2 + y, -z + 1]

**Fig. 2.**
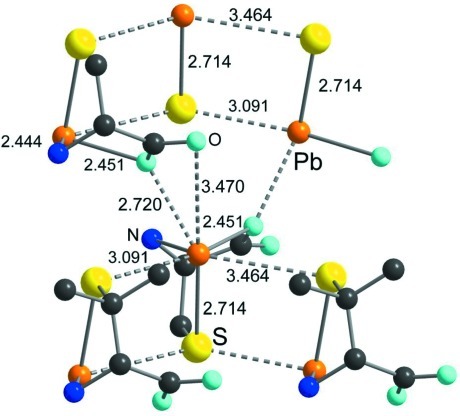
A perspective drawing of the title compound showing coordination geometry around Pb and double bridges formed by sulfur atoms giving rise to infinite chains held together via bridging carboxylate groups. H atoms were omitted for clarity.

**Fig. 3.**
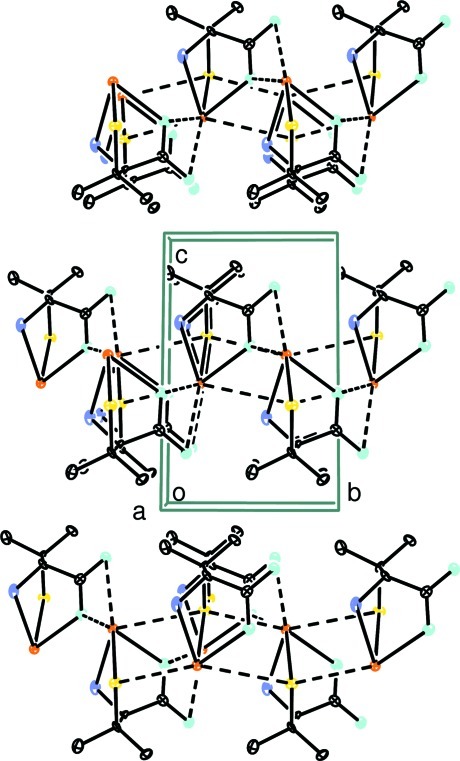
A view of the unit cell of the crystal structure of the title compound. H atoms non-participating in hydrogen-bonding were omitted for clarity. Long bond distances are drawn as dotted lines.

**Fig. 4.**
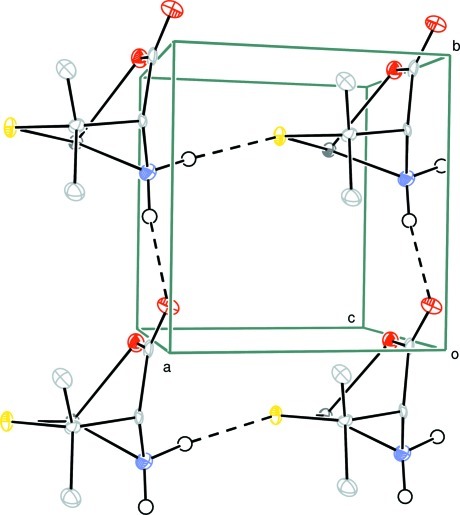
A view of the N—-H···O and N—H···S hydrogen bonds (dotted lines) in the crystal structure of the title compound. H atoms were omitted for clarity.

## supplementary crystallographic information

### Comment

Lead is a serious environmental contaminant. The extensive use of lead as metal and in lead compounds into modern times, *e. g.*, in alkyl lead additives in leaded gasoline, battery manufacturing and in paints, has made lead a ubiquitous pollutant in the ecosystem. The soluble Pb^II^ ion with its 5 d^10^ 6 s^2^ electronic configuration in the valence shell has a very flexible coordination behaviour. It is a neurotoxic heavy metal ion that perturbs multiple enzyme systems affecting areas of the brain that regulate behavior and nerve cell development and any site with sulfhydryl groups is vulnerable (Needleman, 2004). In particular Zn(II) can be replaced in enzymes, *e. g.*, inhibiting the heme biosynthetic pathway, even though the effective ionic radius in four-coordination of the soft Pb^II^ ion (0.98 Å) is significantly larger than that of Zn^II^ (0.60 Å). Pb^II^ can also adapt to replace Ca(II) in bone (Bressler *et al.*, 1999; Godwin, 2001).

Treatments of lead(II) poisoning are mainly based on using chelators that form strong bonds to heavy metal ions, such as the disodium salt of the calcium edta complex (CaNa_2_edta) and dimercaprol (BAL), which are injected, and DMSA (*meso*-2, 3-dimercaptosuccinic acid) and *D*-penicillamine (H_2_Pen), which are administered orally (Sinicropi *et al.*, 2010; Casas & Sordo, 2006).

The binding of Pb^II^ to the tridentate chelator H_2_Pen containing a sulfhydryl group is of interest for better understanding of the coordination behaviour in biological systems and for the design of specific detoxifying agents. The coordination geometry around the Pb^II^ ion in the crystalline title compound (PbPen), which precipitates in a wide pH range from penicillamine solutions containing lead(II) ions, was previously discussed by Freeman *et al.*, (1974). However, the atomic coordinates of the crystal structure were not reported, nor deposited in the Cambridge Structural Database (refcode: DPENPB]. Here, we report the crystal structure of PbPen, and also discuss the Pb—Pb distances in this polymeric structure with double bridged thiolate chains.

Mixing Pb(NO_3_)_2_ and *D*-penicillamine in 1:2 molar ratio resulted in a 1:1 complex, PbPen, formed in an alkaline solution. The ligand is coordinated to the Pb^II^ ion in a tridentate mode: Pb—N 2.444 (9) Å, Pb—O 2.451 (7) Å and Pb—S 2.714 (2) Å (Fig. 1). The sulfur atom acts as a bridge with Pb—S distances of 3.091 (2) and 3.464 (2) Å to two other neighbouring Pb^II^ ions located at 4.363 Å relative to the original Pb^II^ ion, forming a double thiolate chain in a polymeric structure. Moreover, the coordinated carboxylate oxygen atom forms bridges to the lead ions (Pb—O 2.720 (7) Å) in the adjacent chain with two Pb^II^ ions at 4.663 Å relative to the central Pb^II^ ion (Fig. 2). The coordination sphere of lead can be described as a distorted pentagonal bipyramid if the Pb—O interactions to the carboxylate oxygen atoms are considered as axial interaction opposite the short Pb—S bond (2.714 (2) Å), and also including a possible stereochemically active inert electron pair in the void in the equatorial plane between the two long Pb—S interactions, 3.091 (2) Å and 3.464 (2) Å (Fig. 3) (Freeman *et al.*, 1974).

The amino H-atoms of the title complex are hydrogen bonded to a S-atom (N1—H1B···S1) along the *a*-axis and an O-atom (N1—H1A···O2) along the *b*-axis resulting in a cluster of four complex units giving rise to a 16-membered ring in the *ab*-plane which can be best described as a *R*_4_^4^(16) motif in the graph set notation (Bernstein *et al.*, 1994) (Fig. 4).

### Experimental

To a solution of 2 mmol penicillamine (H_2_Pen) in boiled O_2_-free water, 1 mmol Pb(NO_3_)_2_ was added, forming a white precipitate, which was dissolved by adding 2 and 6 *M* NaOH solution, increasing the pH to 11.2; [Pb^2+^] = 0.1 *M*. After several days in the refrigerator colorless plates formed.

### Refinement

All H atoms were positioned geometrically and refined using a riding model, with N—H = 0.92 Å and C—H = 0.98 and 1.00 Å, for methyl and methylene H-atoms, respectively, and the *U*_iso_(H) were allowed at 1.5*U*_eq_(N/C). An absolute structure was determined using 966 Friedel pairs of reflections which were not merged; the Flack parameter was 0.03 (2) (Flack, 1983). The largest residual peaks in the final difference map were located in the close proximity of the Pb atom and may be attributed to inadequate absorption correction.

### Crystal data

**Table:**

[Pb(C_5_H_9_NO_2_S)]	*F*(000) = 320
*M**_r_* = 354.38	*D*_x_ = 3.118 Mg m^−^^3^
Monoclinic, *P*2_1_	Mo *K*α radiation, λ = 0.71073 Å
Hall symbol: P 2yb	Cell parameters from 2175 reflections
*a* = 6.251 (4) Å	θ = 3.4–30.0°
*b* = 6.179 (3) Å	µ = 22.56 mm^−^^1^
*c* = 10.259 (6) Å	*T* = 123 K
β = 107.72 (2)°	Plate, colorless
*V* = 377.5 (4) Å^3^	0.06 × 0.05 × 0.02 mm
*Z* = 2	

### Data collection

**Table:**

Nonius KappaCCD diffractometer	2157 independent reflections
Radiation source: fine-focus sealed tube	2027 reflections with *I* > 2σ(*I*)
Graphite monochromator	*R*_int_ = 0.059
ω and φ scans	θ_max_ = 30.0°, θ_min_ = 3.4°
Absorption correction: multi-scan (*SORTAV*; Blessing, 1997)	*h* = −8→8
*T*_min_ = 0.345, *T*_max_ = 0.661	*k* = −8→8
6589 measured reflections	*l* = −14→14

### Refinement

**Table:**

Refinement on *F*^2^	Secondary atom site location: difference Fourier map
Least-squares matrix: full	Hydrogen site location: inferred from neighbouring sites
*R*[*F*^2^ > 2σ(*F*^2^)] = 0.032	H-atom parameters constrained
*wR*(*F*^2^) = 0.085	*w* = 1/[σ^2^(*F*_o_^2^) + (0.0541*P*)^2^ + 1.7279*P*] where *P* = (*F*_o_^2^ + 2*F*_c_^2^)/3
*S* = 1.09	(Δ/σ)_max_ < 0.001
2157 reflections	Δρ_max_ = 2.76 e Å^−^^3^
93 parameters	Δρ_min_ = −3.17 e Å^−^^3^
1 restraint	Absolute structure: Flack (1983), 966 Friedel pairs
Primary atom site location: structure-invariant direct methods	Flack parameter: 0.03 (2)

### Special details

**Table:**

Geometry. All e.s.d.'s (except the e.s.d. in the dihedral angle between two l.s. planes) are estimated using the full covariance matrix. The cell e.s.d.'s are taken into account individually in the estimation of e.s.d.'s in distances, angles and torsion angles; correlations between e.s.d.'s in cell parameters are only used when they are defined by crystal symmetry. An approximate (isotropic) treatment of cell e.s.d.'s is used for estimating e.s.d.'s involving l.s. planes.
Refinement. Refinement of *F*^2^ against ALL reflections. The weighted *R*-factor *wR* and goodness of fit *S* are based on *F*^2^, conventional *R*-factors *R* are based on *F*, with *F* set to zero for negative *F*^2^. The threshold expression of *F*^2^ > σ(*F*^2^) is used only for calculating *R*-factors(gt) *etc*. and is not relevant to the choice of reflections for refinement. *R*-factors based on *F*^2^ are statistically about twice as large as those based on *F*, and *R*- factors based on ALL data will be even larger.

### Fractional atomic coordinates and isotropic or equivalent isotropic displacement parameters (Å^2^)

**Table:**

	*x*	*y*	*z*	*U*_iso_*/*U*_eq_	
Pb1	0.70681 (4)	0.20419 (12)	0.44819 (2)	0.01059 (9)	
S1	0.4620 (3)	0.2363 (4)	0.6251 (2)	0.0123 (5)	
O1	0.9300 (11)	0.4969 (11)	0.5855 (7)	0.0145 (13)	
O2	1.0202 (11)	0.6382 (11)	0.7971 (7)	0.0162 (13)	
N1	0.9562 (13)	0.0937 (13)	0.6716 (9)	0.0152 (16)	
H1A	0.9180	−0.0416	0.6949	0.018*	
H1B	1.1032	0.0912	0.6715	0.018*	
C1	0.6876 (13)	0.2355 (15)	0.7921 (9)	0.0103 (18)	
C2	0.9226 (13)	0.2620 (12)	0.7695 (9)	0.0091 (16)	
H2	1.0399	0.2389	0.8596	0.011*	
C3	0.9615 (14)	0.4861 (15)	0.7162 (9)	0.0106 (16)	
C4	0.645 (2)	0.410 (2)	0.8842 (13)	0.014 (2)	
H4A	0.7584	0.3999	0.9743	0.017*	
H4B	0.4954	0.3902	0.8940	0.017*	
H4C	0.6544	0.5519	0.8441	0.017*	
C5	0.680 (2)	0.016 (2)	0.8594 (14)	0.018 (2)	
H5A	0.8064	0.0049	0.9436	0.021*	
H5B	0.6895	−0.0998	0.7964	0.021*	
H5C	0.5384	0.0031	0.8816	0.021*	

### Atomic displacement parameters (Å^2^)

**Table:**

	*U*^11^	*U*^22^	*U*^33^	*U*^12^	*U*^13^	*U*^23^
Pb1	0.00940 (13)	0.01069 (14)	0.01203 (14)	0.0004 (2)	0.00381 (9)	0.0000 (2)
S1	0.0067 (7)	0.0167 (15)	0.0139 (8)	0.0002 (8)	0.0039 (6)	−0.0002 (9)
O1	0.013 (3)	0.016 (3)	0.015 (3)	−0.001 (2)	0.005 (2)	0.002 (3)
O2	0.021 (3)	0.012 (3)	0.016 (3)	−0.005 (2)	0.006 (3)	−0.002 (2)
N1	0.011 (3)	0.011 (4)	0.025 (4)	0.003 (3)	0.008 (3)	0.001 (3)
C1	0.012 (3)	0.008 (5)	0.012 (3)	0.001 (3)	0.005 (3)	0.005 (3)
C2	0.004 (3)	0.010 (4)	0.011 (4)	0.000 (2)	−0.001 (3)	−0.006 (3)
C3	0.003 (3)	0.014 (4)	0.015 (4)	0.000 (3)	0.002 (3)	0.003 (3)
C4	0.015 (5)	0.016 (5)	0.014 (5)	−0.002 (4)	0.009 (4)	−0.006 (4)
C5	0.017 (5)	0.015 (5)	0.025 (6)	−0.002 (4)	0.011 (4)	−0.003 (4)

### Geometric parameters (Å, º)

**Table:**

Pb1—N1	2.444 (9)	N1—H1B	0.9200
Pb1—O1	2.451 (7)	C1—C4	1.507 (15)
Pb1—S1	2.714 (2)	C1—C5	1.528 (16)
Pb1—O1^i^	2.719 (7)	C1—C2	1.564 (11)
Pb1—S1^ii^	3.091 (3)	C2—C3	1.535 (12)
Pb1—S1^iii^	3.465 (3)	C2—H2	1.0000
S1—Pb1^iii^	3.091 (3)	C4—H4A	0.9800
S1—C1	1.858 (9)	C4—H4B	0.9800
O1—C3	1.297 (11)	C4—H4C	0.9800
O1—Pb1^iv^	2.719 (7)	C5—H5A	0.9800
O2—C3	1.233 (11)	C5—H5B	0.9800
N1—C2	1.504 (11)	C5—H5C	0.9800
N1—H1A	0.9200		
			
N1—Pb1—O1	65.0 (3)	C4—C1—S1	110.3 (7)
N1—Pb1—S1	73.8 (2)	C5—C1—S1	107.4 (7)
O1—Pb1—S1	84.25 (17)	C2—C1—S1	110.3 (6)
N1—Pb1—O1^i^	70.7 (2)	N1—C2—C3	108.5 (7)
O1—Pb1—O1^i^	93.98 (13)	N1—C2—C1	110.8 (7)
S1—Pb1—O1^i^	141.45 (16)	C3—C2—C1	113.9 (7)
N1—Pb1—S1^ii^	92.28 (19)	N1—C2—H2	107.8
O1—Pb1—S1^ii^	157.30 (16)	C3—C2—H2	107.8
S1—Pb1—S1^ii^	90.61 (6)	C1—C2—H2	107.8
O1^i^—Pb1—S1^ii^	76.40 (15)	O2—C3—O1	125.2 (9)
C1—S1—Pb1	101.1 (3)	O2—C3—C2	119.7 (8)
C1—S1—Pb1^iii^	109.5 (3)	O1—C3—C2	115.1 (8)
Pb1—S1—Pb1^iii^	97.24 (7)	C1—C4—H4A	109.5
C3—O1—Pb1	115.9 (6)	C1—C4—H4B	109.5
C3—O1—Pb1^iv^	106.6 (5)	H4A—C4—H4B	109.5
Pb1—O1—Pb1^iv^	128.8 (3)	C1—C4—H4C	109.5
C2—N1—Pb1	104.7 (5)	H4A—C4—H4C	109.5
C2—N1—H1A	110.8	H4B—C4—H4C	109.5
Pb1—N1—H1A	110.8	C1—C5—H5A	109.5
C2—N1—H1B	110.8	C1—C5—H5B	109.5
Pb1—N1—H1B	110.8	H5A—C5—H5B	109.5
H1A—N1—H1B	108.9	C1—C5—H5C	109.5
C4—C1—C5	108.3 (8)	H5A—C5—H5C	109.5
C4—C1—C2	111.7 (8)	H5B—C5—H5C	109.5
C5—C1—C2	108.7 (8)		
			
N1—Pb1—S1—C1	17.3 (4)	Pb1^iii^—S1—C1—C4	32.8 (7)
O1—Pb1—S1—C1	−48.4 (4)	Pb1—S1—C1—C5	−107.5 (6)
O1^i^—Pb1—S1—C1	40.8 (4)	Pb1^iii^—S1—C1—C5	150.6 (6)
S1^ii^—Pb1—S1—C1	109.5 (3)	Pb1—S1—C1—C2	10.8 (6)
N1—Pb1—S1—Pb1^iii^	128.8 (2)	Pb1^iii^—S1—C1—C2	−91.1 (6)
O1—Pb1—S1—Pb1^iii^	63.19 (17)	Pb1—N1—C2—C3	−53.8 (7)
O1^i^—Pb1—S1—Pb1^iii^	152.4 (2)	Pb1—N1—C2—C1	71.9 (7)
S1^ii^—Pb1—S1—Pb1^iii^	−138.97 (9)	C4—C1—C2—N1	−177.1 (8)
N1—Pb1—O1—C3	−36.8 (6)	C5—C1—C2—N1	63.5 (10)
S1—Pb1—O1—C3	38.0 (6)	S1—C1—C2—N1	−54.0 (8)
O1^i^—Pb1—O1—C3	−103.4 (5)	C4—C1—C2—C3	−54.5 (10)
S1^ii^—Pb1—O1—C3	−39.7 (8)	C5—C1—C2—C3	−173.9 (8)
N1—Pb1—O1—Pb1^iv^	106.1 (4)	S1—C1—C2—C3	68.6 (8)
S1—Pb1—O1—Pb1^iv^	−179.1 (3)	Pb1—O1—C3—O2	−161.7 (7)
O1^i^—Pb1—O1—Pb1^iv^	39.6 (3)	Pb1^iv^—O1—C3—O2	47.6 (10)
S1^ii^—Pb1—O1—Pb1^iv^	103.2 (4)	Pb1—O1—C3—C2	18.9 (9)
O1—Pb1—N1—C2	45.4 (5)	Pb1^iv^—O1—C3—C2	−131.8 (6)
S1—Pb1—N1—C2	−45.8 (5)	N1—C2—C3—O2	−154.8 (8)
O1^i^—Pb1—N1—C2	149.5 (5)	C1—C2—C3—O2	81.3 (10)
S1^ii^—Pb1—N1—C2	−135.8 (5)	N1—C2—C3—O1	24.6 (9)
Pb1—S1—C1—C4	134.7 (7)	C1—C2—C3—O1	−99.3 (8)

Symmetry codes: (i) −*x*+2, *y*−1/2, −*z*+1; (ii) −*x*+1, *y*−1/2, −*z*+1; (iii) −*x*+1, *y*+1/2, −*z*+1; (iv) −*x*+2, *y*+1/2, −*z*+1.

### Hydrogen-bond geometry (Å, º)

**Table:**

*D*—H···*A*	*D*—H	H···*A*	*D*···*A*	*D*—H···*A*
N1—H1*B*···S1^v^	0.92	2.59	3.453 (8)	156
N1—H1*A*···O2^vi^	0.92	2.24	3.070 (10)	150

Symmetry codes: (v) *x*+1, *y*, *z*; (vi) *x*, *y*−1, *z*.
